# The Role of Calcium-Permeable Kainate and AMPA Receptors in the Leading Reaction of GABAergic Neurons to Excitation

**DOI:** 10.3390/cimb48010082

**Published:** 2026-01-14

**Authors:** Valery P. Zinchenko, Artem M. Kosenkov, Alex I. Sergeev, Fedor V. Tyurin, Egor A. Turovsky, Bakytzhan K. Kairat, Arailym E. Malibayeva, Gulmira A. Tussupbekova, Sultan T. Tuleukhanov

**Affiliations:** 1 Federal Research Center “Pushchino Scientific Center for Biological Research of the Russian Academy of Sciences”, Institute of Cell Biophysics of the Russian Academy of Sciences, Pushchino 142290, Russiasergeev.bio@gmail.com (A.I.S.); turovsky.84@mail.ru (E.A.T.); 2 Faculty of Biology and Biotechnology, Al-Farabi Kazakh National University, Almaty 050040, Kazakhstansultan.tuleuhanov@kaznu.edu.kz (S.T.T.)

**Keywords:** early signal of GABAergic neurons, calcium-permeable СР-КА and СР-АМРА receptors, low-threshold spiking (LTS) interneurons, Ca^2+^ imaging

## Abstract

Excitable neurons are intrinsically capable of firing action potentials (AP), yet a state of hyperexcitability is prevented in the central nervous system by powerful GABAergic inhibition. For this inhibition to be effective, it must occur before excitatory signals can initiate runaway activity, implying the existence of a proactive control system. To test for such proactive inhibition, we used Ca^2+^ imaging and patch-clamp recording to measure how hippocampal neurons respond to depolarization and glutamatergic agonists. In mature hippocampal cultures (14 days in vitro (DIV)) and acute brain slices from two-month-old rats, neurons exhibited non-simultaneous responses to various excitatory stimuli, including KCl, NH_4_Cl, forskolin, domoic acid, and glutamate. We observed that the Ca^2+^ rise occurred significantly earlier in GABAergic neurons than in glutamatergic neurons. This delay in glutamatergic neurons was abolished by GABA(A) receptor inhibitors, suggesting a mechanism of preliminary γ-aminobutyric acid (GABA) release. We further found that these early-responding GABAergic neurons express calcium-permeable kainate and AMPA receptors (CP-KARs and CP-AMPARs). Application of domoic acid induced an immediate Ca^2+^ increase in neurons expressing these receptors, but a delayed response in others. Crucially, when domoic acid was applied in the presence of the AMPA receptor inhibitors NBQX or GYKI-52466, the response delay in glutamatergic neurons was significantly prolonged. This confirms that CP-KARs on GABAergic neurons are responsible for the delayed excitation of glutamatergic neurons. In hippocampal slices from two-month-old rats, depolarization with 50 mM KCl revealed two distinct neuronal populations based on their calcium dynamics: a majority group (presumably glutamatergic) exhibited fluctuating Ca^2+^ signals, while a minority (presumably GABAergic) showed a steady, advancing increase in [Ca^2+^]i. This distinction was reinforced by the application of domoic acid. The “advancing-response” neurons reacted to domoic acid with a similar prompt increase, whereas the “fluctuating-response” neurons displayed an even more delayed and fluctuating reaction (80 s delay). Therefore, we identify a subgroup of hippocampal neurons—in both slices and cultures—that respond to depolarization and domoic acid with an early [Ca^2+^]_i_ signal. Consistent with our data from cultures, we conclude these early-responding neurons are GABAergic. Their early GABA release directly explains the delayed Ca^2+^ response observed in glutamatergic neurons. We propose that this proactive mechanism, mediated by CP-KARs on GABAergic neurons, is a primary means of protecting the network from hyperexcitation. Furthermore, the activity of these CP-KAR-expressing neurons is itself regulated by GABAergic neurons containing CP-AMPARs.

## 1. Introduction

This article presents new data demonstrating that GABAergic neurons expressing calcium-permeable kainate and AMPA receptors (CP-KARs and CP-AMPARs) generate an anticipatory response to diverse forms of hyperexcitation. Previous research has established that CP-KARs and CP-AMPARs are predominantly expressed in hippocampal GABAergic neurons of adult rats [1,2]. These receptors define two major subgroups that together constitute over 55% of the GABAergic population [1]: one group expresses CP-KARs, and the other expresses CP-AMPARs [1,3,4]. Anatomically, CP-AMPAR-expressing GABAergic neurons innervate those expressing CP-KARs [1,5]. Functionally, these receptors act as low-threshold channels, enabling neuronal excitation by weak depolarizations or low doses of agonists like domoic acid (DoA) [1,3]. Agonists of CP-KARs, such as ATPA or SYM2081, selectively increase intracellular calcium levels in CP-KAR-expressing neurons, induce γ-aminobutyric acid (GABA) release, and consequently suppress downstream neuronal activity [5,6,7]. These properties have been leveraged to develop methods for visualizing these specific neurons in cultures [2,3]. Notably, during DoA exposure, these GABAergic neurons exhibit a unique response: rather than being fully excited to fire AP and Ca^2+^ oscillations, the calcium influx through CP-KARs reaches a subthreshold level that is sufficient to trigger GABA release directly [6].

This functional profile aligns with that of low-threshold spiking (LTS) interneurons, which are known to induce advanced inhibition and are excited by small shifts in membrane potential [8,9]. While proactive reaction in LTS interneurons is often attributed to T-type calcium channels, the CP-KAR-mediated mechanism described here provides an alternative pathway. It is important to note the following discrepancy in population size: while LTS interneurons are estimated to comprise 4.5–15% of hippocampal GABAergic neurons [10], our data indicate that over 55% express CP-KARs or CP-AMPARs in cultures [1]. This evidence suggests that these low-threshold receptor phenotypes may be more widespread among GABA neurons than the defined LTS interneuron subtype.

This study demonstrates that GABAergic neurons expressing CP-KARs and CP-AMPARs respond earlier than glutamatergic neurons to both depolarization and glutamate receptor agonists in slices of the hippocampus from adult two-month-old animals. Thus, the mechanisms of the preliminary reaction of GABAergic neurons and the delay in the excitation of other neurons are realized in the brains of adult animals.

## 2. Materials and Methods

### 2.1. Cell Culture Preparation

Primary neuronal–glial co-cultures were prepared from the hippocampi of neonatal Wistar rats (postnatal days 0–2). Briefly, after decapitation, hippocampal tissue was rapidly dissected in cold Versene solution, minced, and digested with 1% trypsin for 10 min at 37 °C under continuous agitation (~500 rpm). The digested tissue was then washed with chilled Neurobasal medium and mechanically dissociated via gentle trituration. The cell suspension was centrifuged at 2000 rpm for 3 min, and the pellet was resuspended in culture medium (neurobasal medium supplemented with 2% B27, 0.5 mM glutamine, and 1:250 penicillin–streptomycin). To optimize the conditions for postnatal neurons, the NaCl concentration was adjusted to 4 g/L, matching that of the Neurobasal-A medium.

For plating, 100 μL aliquots of the cell suspension were deposited in glass cylinders (inner diameter, 6 mm; height, 7 mm) positioned on polyethyleneimine-coated glass coverslips in Petri dishes. The cultures were incubated for 40 min (37 °C, 5% CO_2_, and 95% humidity) to allow for cell attachment; subsequently, the cylinders were carefully removed, and 2 mL of complete medium was added to each dish. Cultures were maintained with partial medium replacement (one-third volume) every four days and used for experiments after 13–14 DIV.

### 2.2. Hippocampal Slices

Hippocampal slices were obtained from two-month-old male Sprague-Dawley rats. The rats were subjected to terminal anesthesia via inhalation overdose of halothane. Then, the brain was extracted and placed in ice-cold artificial cerebrospinal medium (aCSF) with the following composition: 124 mM NaCl, 26 mM NaHCO_3_, 3 mM KCl, 2 mM CaCl_2_, 1.25 mM NaH_2_PO_4_, 1 mM MgSO_4_, and 10 mM glucose, saturated with a mixture of 95% O_2_ and 5% CO_2_ (pH 7.4) with 9 mM Mg^2+^.

Coronal slices of the CA1 region of the hippocampus, measuring 300 μm thick, were obtained using a Leica VT1200 vibratome (Leica Biosystems, Deer Park, IL, USA). The obtained slices were incubated for 1 h at room temperature in standard aCSF, which was saturated with a mixture of 95% O_2_ and 5% CO_2_.

Subsequently, the slices were transferred to a perfusion chamber and loaded with the fluorescent Ca^2+^-sensitive probe Fluo-4 AM at a final concentration of 5 μM with the addition of 0.02% Pluronic F-127 for 1 h. To complete the de-esterification of the probe, the slices were washed for 15 min in a perfusion system with aCSF saturated with a mixture of 95% O_2_ and 5% CO_2_.

After loading with the fluorescent probe, the slices were transferred to a specialized chamber for visualization—an RC-26G Open Diamond Bath Imaging Chamber (Warner Instruments, Holliston, MA, USA)—and placed on the stage of a Zeiss LSM 510 Meta confocal laser-scanning microscope (Carl Zeiss, Oberkochen, Germany). An Achroplan 40×/0.8 W objective was used. The microscope settings for excitation and registration of Fluo-4 fluorescence were as follows: argon laser (488 nm), an HFT 488 dichroic mirror, a BP 500–550 nm emission filter, a gain of 600–800, and an offset of 0.1. The acquired image series was analyzed using ImageJ software (v1.54p, RRID: SCR_003070).

### 2.3. Fluorescent [Ca^2+^]_i_ Measurements

Intracellular calcium dynamics were monitored using the ratiometric indicator Fura-2 AM. Hippocampal cultures were incubated with 2–3 μM of the dye for 40 min at 28–37 °C in Hank’s balanced salt solution (HBSS) containing (in mM) 136 NaCl, 3 KCl, 0.8 MgSO_4_, 1.25 KH_2_PO_4_, 0.35 Na_2_HPO_4_, 1.4 CaCl_2_, 10 HEPES, and 10 glucose, adjusted to pH 7.35. After loading, cells were washed several times with fresh HBSS to remove excess probe. Fluorescence was recorded using an inverted epifluorescence microscope (Leica DMI6000B, Leica Microsystems, Wetzlar, Germany) equipped with a Hamamatsu CCD camera and an external excitation filter wheel. Sequential illumination at 340 and 387 nm was provided, and emission data were collected at 510 ± 40 nm using the FU2 filter set. Time-lapse imaging was performed at 28–30 °C in HBSS. Calcium signals were analyzed by calculating the 340/387 fluorescence ratio from regions of interest drawn over neuronal somata. Background signals obtained from cell-free areas of the field were subtracted before ratio calculation. The resulting traces represent relative changes in intracellular Ca^2+^ concentrations. In each experiment, *N* = 125–250 neurons were analyzed in the field of view in three in n = 3–5 replicates.

### 2.4. Electrophysiological Measurements

Experiments using the patch-clamp technique in whole-cell configuration were carried out on a fluorescent station with built-in microincubator and electrophysiological patchclamp setup equipped with a Hamamatsu ORCA-Flash camera (Hamamatsu Photonics K.K., Shizuoka, Japan). Electrophysiological characteristics of neurons were recorded at 28 °C using an Axopatch 200B amplifier (Axon Instruments, Union City, CA, USA). Data were digitized using a low-noise data acquisition system (Axon DigiData 1440A digital co1nverter) (Molecular Devices, San Jose, CA, USA) with pCLAMP 10 software from Axon Instruments.

Patch pipettes were filled with an internal solution of the following composition (in mM): 5 KCl, 130 K-gluconate, 1 MgCl_2_ × 6H_2_O, 0.25 EGTA, 4 HEPES, 2 Na_2_-ATP, 0.3 Mg-ATP, 0.3 Na-GTP, and 10 Na_2_-phosphocreatine. The osmolarity of the solution was adjusted to 305–310 mOsm, and the pH was set to 7.2.

For all recordings, we used an extracellular medium containing (in mM): 156 NaCl, 3 KCl, 2 MgSO_4_, 0.35 Na_2_HPO_4_, 1.25 KH_2_PO_4_, 1.4–1.5 CaCl_2_, 10 glucose, and 10 HEPES. The pH of the bath solution was maintained at 7.35–7.4.

### 2.5. Reagents

The following reagents were used: domoic acid, NASPM trihydrochloride, ATPA, NBQX, forskolin (Tocris Bioscience, Bristol, UK), bicuculline (Cayman Chemical, Ann Arbor, MI, USA), L-Glutamic acid (Sigma-Aldrich, Saint Louis, MO, USA), NH_4_Cl (AppliChem, Darmstadt, Germany), Fura-2 AM (Molecular Probes, Eugene, OR, USA), Neurobasal-A medium (Life Technologies, Grand Island, NY, USA), B-27 supplement (Life Technologies, Grand Island, NY, USA), and Trypsin 2.5% (Life Technologies, Grand Island, NY, USA). Statistical and data analysis ImageJ software (National Institutes of Health, Bethesda, MD, USA) was used for image analysis. The results are presented as single-cell signals or the average cell signal per the field of view ± standard error (SE). Origin Pro 2021 version 9.8.0.200 was used for graph creation and analysis (OriginLab, Northampton, MA, USA). Electrophysiological data were analyzed using ClampFit 10 software (Molecular Devices, San Jose, CA, USA). All experiments were performed using the cultures from at least 2 to 3 different animals. The percentage of neurons that responded to ATPA in the field of view varied from 5% to 15%.

## 3. Results

### 3.1. GABAergic Neurons Expressing CP-KARs and CP-AMPARs Receptors

**Figure 1:**
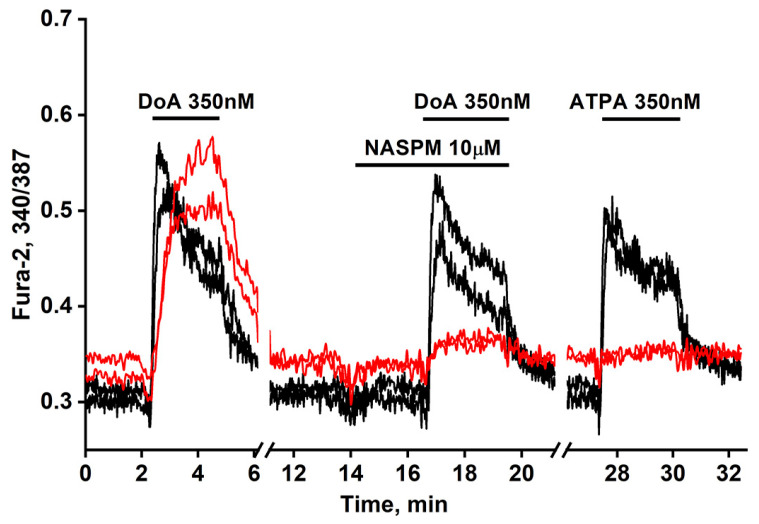
Dynamic of [Ca^2+^]_i_ changes in two GABAergic neurons expressing CP-AMPARs (red curves) and in two expressing CP-KARs (black curves). Both subtypes respond to domoic acid (DoA) (350 nM). In one of the subtypes responding more slowly to DoA, the response is inhibited by NASPM (10 μM), while the other, responding more quickly, is activated by ATPA (350 nM). *N* = 125, n = 3.

We previously established simple methods of visualizing distinct populations of GABAergic neurons [2,3]. To identify neurons expressing CP-KARs, we recorded an increase in [Ca^2+^]_i_ levels following application of the selective agonist (RS)-2-amino-3-(3-hydroxy-5-tertbutylisoxazol-4-yl) propanoic acid (ATPA). To identify neurons expressing CP-AMPARs, we apply domoic acid and then block the subsequent Ca^2+^ signal with the selective antagonist NASPM (Figure 1). (In the presence of other Ca^2+^ channels’ inhibitors.

Figure 1 illustrates a representative experiment tracking changes in [Ca^2+^]_i_ in two GABAergic neuron subtypes: those expressing CP-KARs (black curves) and those expressing CP-AMPARs (red curves). Application of domoic acid (DoA), an agonist for both receptors, increased [Ca^2+^]_i_ in both subtypes. The response was faster in ATPA-sensitive neurons (CP-KARs) and slower in NASPM-sensitive neurons (CP-AMPARs). The subtype-specificity of the responses was confirmed by applying NASPM, which inhibited the DoA-induced Ca^2+^ signal in CP-AMPAR-expressing neurons, and ATPA, which selectively induced a [Ca^2+^]_i_ rise in CP-KAR-expressing neurons. Immunostaining verified that both neuronal subtypes are GABAergic [1,2]. This functional distinction is complemented by the known subcellular localization of these receptors: CP-KARs are primarily presynaptic [11,12,13], while CP-AMPARs are postsynaptic [11,14,15,16]. While our prior work revealed an anticipatory Ca^2+^ response to DoA and NH_4_Cl in GABAergic neurons expressing CP-KARs and CP-AMPARs [1,5], the precise mechanism remained unclear. In this study, we investigate the cellular basis of this phenomenon.

### 3.2. The Advancing Response of Neurons to DoA Is Eliminated by GABA(A) Receptor Inhibitors

**Figure 2:**
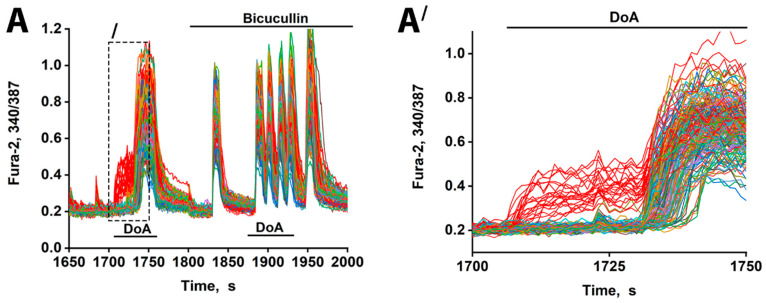
Changes in [Ca^2+^]i in neurons in response to 100 nM DoA in the control and in the presence of 10 µM bicuculline. (**A**) The delay in the response of glutamatergic neurons to DoA is negated by bicuculline. Between 23 and 33 out of 200 cells responded with an early signal (red curves). (**A′**) This is the left part of (**A**) (indicated by dashed lines). The delay is 30 ± 5 s. GABAergic neurons (red curves) desynchronize other neurons (other colored curves). *N* = 200, n = 3.

Sequential pharmacological manipulation in an oscillating neuronal culture (14 DIV) revealed distinct [Ca^2+^]_i_ dynamics in response to hyperexcitation (Figure 2). Application of DoA, an agonist for both KA and AMPA receptors, induced a biphasic response: an earlier [Ca^2+^]_i_ increase in a small subpopulation of neurons (14% ± 5%; red curves) and a delayed response in the majority.

Consistent with prior studies [1,3], the neurons exhibiting an early response are GABAergic and express CP-KARs and CP-AMPARs. Inhibition of GABA(A) receptors with bicuculline negated the delayed excitation, confirming GABA’s involvement. Bicuculline also increased the amplitude of calcium spikes, an effect attributable to depolarization resulting from postsynaptic Cl^−^ channel closure. The extended timeline (Figure 2A′) further shows that the delayed responses were not synchronous, likely reflecting heterogeneity in the Cl^−^ ion gradient across neurons that influence the membrane potential shift upon the opening of the GABA(A) channel [17,18]. Therefore, beyond simply delaying excitation, GABAergic activity serves to desynchronize the network’s response to hyper-excitatory stimuli. Bicuculline eliminates the heterogeneous output of the GABA(A) receptor network, resulting in synchronized oscillations.

### 3.3. The Excitation Delay Depends on the Concentration of the Agonist and Is Determined by CP-KARs

**Figure 3:**
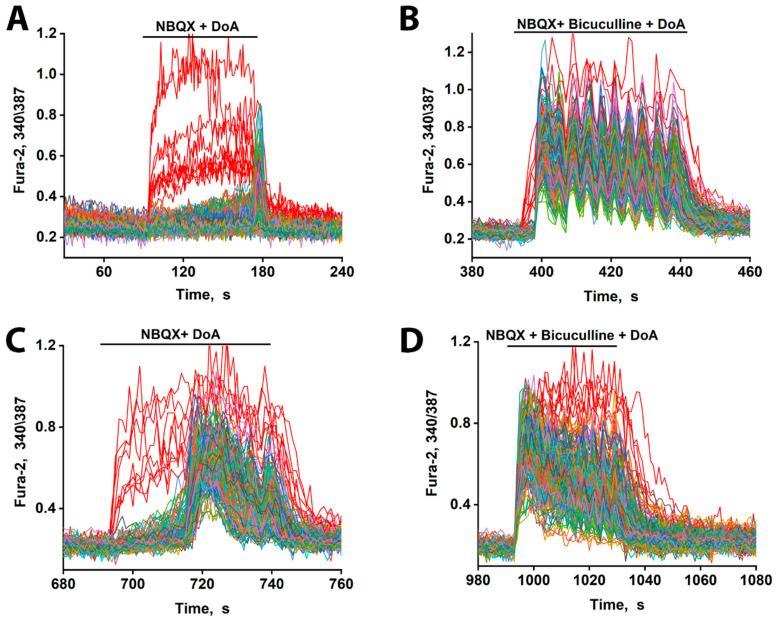
Changes in [Ca^2+^]i in neurons in response to DoA. (**A**) Effect of DoA (100 nM) in the presence of the AMPA receptor inhibitor, 2 µM NBQX. An early response of GABAergic neurons expressing CP-KARs (red curves) is shown. Glutamatergic neurons (gray–blue–green curves) respond with a delay of 80 s. (**B**) Change in [Ca^2+^]i in neurons in response to 100 nM DoA in the presence of 10 µM bicuculline. (**C**) When the DoA concentration is increased to 500 nM, the delay in the response of glutamatergic neurons (gray–blue–green curves) decreases from 80 s to 20 s. (**D**) Bicuculline (10 µM) completely negates the delay in the case of a high concentration of DoA (500 nM). *N* = 200 n = 3.

As we have previously demonstrated, DoA selectively activates kainate receptors (KARs) when AMPARs are blocked by NBQX [1]. Under these conditions, a low concentration of DoA (100 nM) triggered an immediate response in GABAergic neurons expressing CP-KARs. This was followed by a significantly greater delay (~80 s) in the excitation of glutamatergic neurons compared to conditions without NBQX (compare Figure 2 and Figure 3A). This delay was concentration-dependent, as increasing the DoA concentration to 500 nM substantially reduced the latency of glutamatergic excitation, consistent with an increased depolarizing drive that can overcome inhibition (Figure 3C). Furthermore, in the presence of bicuculline, the delay was still evident at the low agonist concentration (Figure 3B) but was completely abolished at the high DoA concentration (Figure 3D).

These results indicate that while both subtypes of GABAergic neurons respond early to DoA, the inhibitory delay in glutamatergic excitation is primarily mediated by the activity of CP-KAR-expressing GABAergic neurons. Our data support the model in which GABAergic neurons expressing CP-AMPARs provide inhibitory control over those expressing CP-KARs, thus modulating the timing of network excitation. Therefore, pharmacological blockade of CP-AMPARs with NBQX disinhibits the CP-KAR-expressing neurons. This disinhibition evidently enhances their GABA release, leading to a more potent and prolonged suppression of their downstream glutamatergic targets.

### 3.4. Early Response of DoA-Sensitive GABAergic Neurons to Glutamate

Unlike NMDA receptors, which require depolarization to relieve Mg^2+^ blocks, CP-AMPARs and CP-KARs permit immediate Ca^2+^ influx without prior depolarization. We therefore hypothesized that neurons expressing these receptors would respond to glutamate more rapidly, particularly at low concentrations.

**Figure 4:**
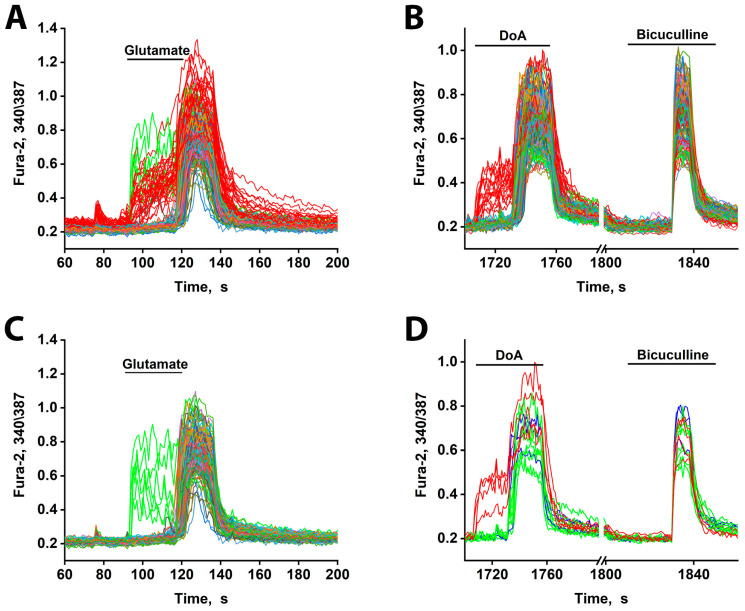
[Ca^2+^]_i_ changes in neurons in response to 10 µM glutamate and DoA. (**A**) The minor subtypes of neurons exhibit an early reaction to glutamate (red and green curves). All DoA-sensitive neurons participate in the early response to glutamate (red curves). The duration of the delay is about 30 s. (**B)** An early response of GABAergic neurons (red curves) to 100 nM DoA. (**C**) A total of 10 µM Glutamate induces a rapid oscillatory response in 3–4% of neurons (green curves), which are not members of the DoA-sensitive neuron subtype. (**D**) The neurons in green (**A**,**C**) respond to DoA with a significant delay. This delay is eliminated by bicuculline. However, during strong depolarization caused by bicuculline, these neurons oscillate synchronously with the other neurons. The data are from a sample of 200 neurons in the field of view. *N* = 200, n = 3.

In mature cultures (14 DIV), glutamate indeed elicited a biphasic Ca^2+^ response: an early response in a minority of neurons and a delayed response in all other neurons (Figure 4A). The early-responding neurons were the same GABAergic cells that responded early to DoA (red curves, Figure 4B) [3].

Additionally, glutamate triggered an early response in a small subset of other neurons (6–8 out of 200) (green curves, Figure 4A,C). These neurons are likely not GABAergic, as they did not respond early to DoA. Instead, they appear to be innervated by the DoA-sensitive GABAergic neurons, as they responded to DoA with a prolonged delay (~3 s longer than average—Figure 4D). This extended delay may indicate a significant Cl^−^ ion gradient in these cells. Alternatively, they might possess a higher affinity for glutamate. Unlike the DoA-sensitive GABAergic neurons, these neurons exhibited asynchronous oscillatory activity upon glutamate application—a pattern characteristic of mild depolarization [19,20].

These results demonstrate that GABAergic neurons expressing CP-KARs and CP-AMPARs are the first to respond to glutamate. This early excitation, consistent with their rapid response kinetics [1,3], delays the firing of their target neurons, thereby desynchronizing network activity.

### 3.5. Early Response of DoA-Sensitive GABAergic Neurons to Depolarization

**Figure 5:**
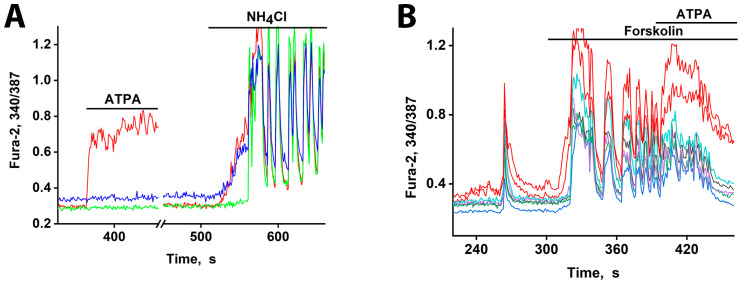
[Ca^2+^]_i_ changes in representative neurons in response to depolarization. (**A**) The response of neurons from three subgroups to depolarization induced by NH_4_Cl (8 mM). An ATPA-sensitive GABAergic neuron (red curve). A GABAergic neuron expressing CP-AMPARs (blue curve). A glutamatergic neuron reacting after a delay of ≈30 s (green curve). Synchronization of oscillations occurs after ≈20 s of general excitation. There are 240 neurons in the field of view. (**B**) An early response to forskolin (70 µM) is observed in neurons that selectively react to ATPA (200 nM) with a [Ca^2+^]_i_ increase (red curves). *N* = 125, n = 2.

GABAergic neurons expressing CP-AMPARs and CP-KARs are poised for rapid activation. These receptors are low-threshold [1,3], and the somata of these neurons receive minimal inhibitory input [2]. Consequently, they can depolarize earlier than other neurons in response to a global stimulus, leading to GABA release [6]. We tested this using moderate depolarization (10–12 mV) induced by 2–8 mM NH_4_Cl, which alters membrane potential by passing through potassium channels [1,21]. In one experiment (Figure 5A), 8 mM NH_4_Cl triggered an immediate [Ca^2+^]_i_ increase in two GABAergic neuron subtypes (red and blue curves) but a delayed response in a glutamatergic neuron (green curve). The glutamatergic population was excited after a 30–40 s delay, and this was followed by synchronous oscillations.

Pharmacological profiling confirmed the identity of the early responders: pretreatment with the CP-KAR (GluK1-containing) agonist ATPA identified one GABAergic subtype (red curve). This finding confirms that both CP-AMPARs and CP-KARs are low-threshold and activate before other calcium channels upon moderate depolarization. The resulting Ca^2+^ influx in these neurons triggers GABA release [5,6], which delays the excitation of their targets. The sharp onset of the delayed signal suggests a cooperative mechanism involving positive feedback.

**Figure 6:**
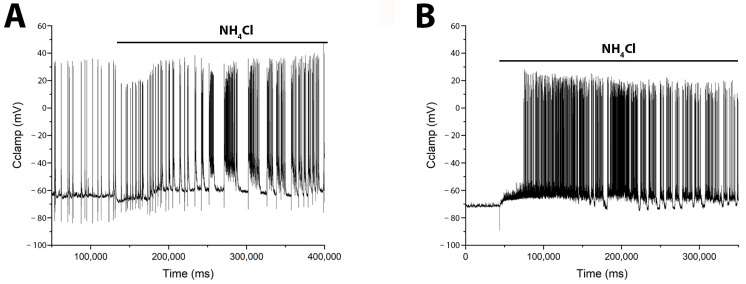
NH_4_Cl (8 mM) induces distinct patterns of burst activity in glutamatergic and GABAergic neurons. Whole-cell patch-clamp recordings from a (**A**) pyramidal glutamatergic neuron *N* = 8, and a (**B**) GABAergic neuron *N* = 3, show the corresponding changes in membrane potential. (**A**) In the glutamatergic neuron, NH_4_Cl elicits an initial hyperpolarization followed by slow depolarization and delayed burst firing. (**B**) In the GABAergic neuron, NH_4_Cl causes an immediate slow depolarization that leads to burst activity. Accordingly, direct electrophysiological measurements revealed a delay in network hyperexcitation following NH_4_Cl application, supporting the model of postsynaptic hyperpolarization in pyramidal cells coupled with slow depolarization in GABAergic neurons.

Previously, we and others showed that the duration and amplitude of Ca^2+^ pulses are directly correlated with the duration and amplitude of AP bursts during epileptiform activity [5,22] (see Supplementary Materials Figure S1). However, to provide direct evidence that GABAergic neurons are activated before pyramidal cells, we performed electrophysiological recordings of membrane potential using the patch-clamp technique in a whole-cell configuration. Figure 6 shows membrane potential changes induced by NH_4_Cl in identified glutamatergic and GABAergic neurons. In glutamate neurons (Figure 6A) NH_4_Cl elicited burst firing after a long delay (≈30 s), beginning with a slight hyperpolarization (≈8 mV) that transitioned into a slow depolarization and subsequent bursts. In contrast, GABAergic neurons (Figure 6B) responded with an immediate slow depolarization, which reached threshold for burst firing after approximately 30 s.

In conclusion, the early response to NH_4_Cl-induced depolarization is mediated by DoA-sensitive GABAergic neurons expressing CP-AMPARs and CP-KARs. However, only the GABAergic neurons expressing CP-KARs are responsible for imposing the excitation delay on glutamatergic neurons, as they provide the direct inhibitory innervation (Figure 3).

### 3.6. GABAergic Neurons Exhibit an Early Response to cAMP Elevation

cAMP typically promotes neuronal excitation by enhancing Na^+^ and Ca^2+^ currents, inhibiting K^+^ currents, and increasing AMPA receptor sensitivity. This leads to depolarization and heightened network oscillations [23,24,25,26]. However, this excitation has a dual effect. In GABAergic interneurons, it enhances inhibitory tone. The increased firing, combined with the relatively slow reuptake of GABA, elevates basal intracellular Ca^2+^ levels, facilitating GABA release and ultimately inhibiting downstream target neurons [5,6].

Our data reveal that this cAMP-mediated excitation does not occur simultaneously across all neurons. Figure 5B demonstrates that GABAergic neurons expressing CP-KARs, identified by their response to ATPA, are the first to respond to forskolin-induced cAMP elevation. The short response delay observed here is likely due to a high baseline excitation level, as indicated by the amplitude of spontaneous synchronous activity and elevated cAMP levels. Consequently, GABAergic neurons expressing CP-KARs and CP-AMPARs are uniquely sensitive, responding rapidly to both direct glutamatergic agonists and moderate depolarizing signals like cAMP.

### 3.7. The Early GABAergic Neuron Response to Excitation in Hippocampal Slices

**Figure 7:**
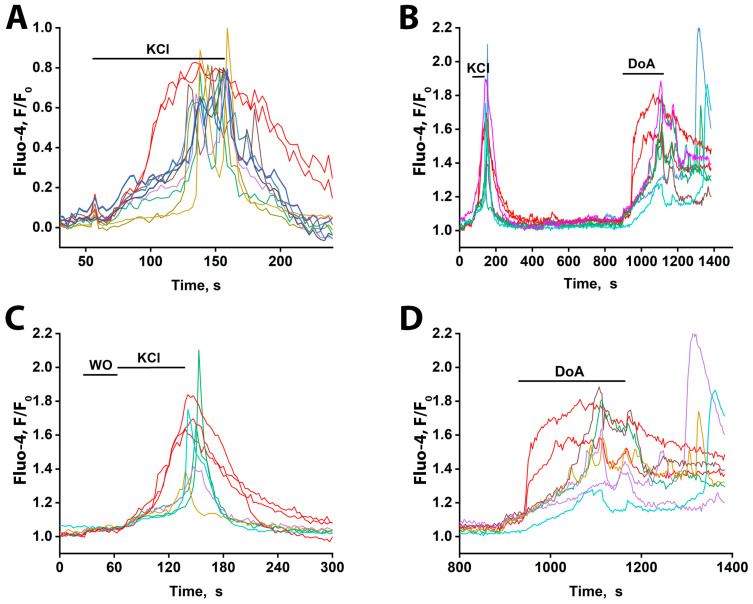
Ca^2+^ dynamics measured with Fluo-4 in slices of the CA1 region of the rat hippocampus in response to 50 mM KCl and 5 µM DoA. (**A**) KCl-induced changes in [Ca^2+^]_i_. Two neurons responded rapidly with a single, non-oscillatory [Ca^2+^]i increase (red traces), while the other neurons reacted after a 30–40 s delay, with oscillatory Ca^2+^ signals. In the field of view, 5 neurons showed the early response, and 50–60 cells showed the delayed, oscillatory response. The average delay between the onset of early and late responses was ≈40 s. (**B**) [Ca^2+^]_i_ dynamics in individual neurons in response to 50 mM KCl and 5 µM DoA. Of the three neurons that responded to KCl with an early signal, two showed a fast response to DoA. (**C**) Time-expanded response to KCl from (**B**). Three neurons responded by increasing their Ca^2+^ levels ≈ 30 s earlier without oscillations. The remaining neurons reacted with a delayed oscillatory response. WO stands for washing out. (**D**) Time-expanded response to 5µM DoA from (**B**). Two neurons responded by increasing their Ca^2+^ levels without oscillations, ≈80 s before the rest of the neurons were activated. The remaining neurons responded with an oscillatory reaction with a delay. Representative curves from two experiments are presented. All neurons that responded quickly (red curves) and 7–5 neurons that responded with oscillations out of 50–60 are presented.

To determine if the early response of GABAergic neurons to excitation also occurs ex vivo, we used hippocampal slices from the CA1 area of two-month-old rats. As shown in Figure 7A, general depolarization with 50 mM KCl elicited distinct response patterns: the majority of neurons displayed oscillatory Ca^2+^ transients (presumed to be glutamatergic—blue-green curves), (see File S1 (avi file)) whereas a small subset exhibited a rapid, non-oscillatory [Ca^2+^]_i_ rise (presumed to be GABAergic—red curves). Response delays among glutamatergic neurons were under 10 s. All responses were reversible upon washout. The oscillatory activity, occurring at approximately 0.2 Hz, is notable, as it matches the frequency of epileptiform activity observed in neuronal cultures.

To pharmacologically validate the identity of the early-responding cells, we applied DoA, an activator of kainate and AMPA receptors (Figure 7B). Consistent with the KCl response, a subset of neurons (three in Figure 7C) displayed an immediate [Ca^2+^]_i_ increase, while the majority responded with delayed oscillations. Subsequent DoA application triggered an early response in two of the three neurons that responded early to KCl (Figure 7B,D), confirming their sensitivity to this glutamatergic agonist and supporting their classification as GABAergic interneurons. The population of neurons that responded to KCl with ≈30 s delayed oscillations also responded to DoA with oscillations, but only after a significantly longer delay (≈80 s). The same neurons are color-coded across panels. One neuron that responded early to KCl did not respond early to DoA, indicating it was likely glutamatergic; a similar phenomenon was also observed in cultured neurons exposed to glutamate and NH_4_Cl (Figure 4).

These results demonstrate that a subset of neurons in hippocampal slices from adult rats, like those in cultures, exhibit an early Ca^2+^ response to both depolarization and the kainate/AMPA receptor agonist DoA. Based on evidence from cell culturing, these rapidly responding cells are GABAergic and express CP-KARs/AMPARs. The observed long latency of the glutamatergic neuronal response in slices is consistent with inhibition from GABA released by these early-responding interneurons. We therefore assume that the early-responding cells in the slices are GABAergic and hypothesize that, as in cultures, their response is mediated by CP-KARs and CP-AMPARs.

## 4. Discussion

This work is devoted to demonstrating the existence of a leading reaction to the excitation of GABAergic neurons in slices of the adult animal hippocampus and investigating the role of GABAergic neurons expressing CP-AMPARs or CP-KARs in the early response to glutamate receptor agonists and depolarization.

Neural circuit stability requires inhibitory neurons to control glutamatergic excitation [27]. For maximum effectiveness, this control should involve a feedforward response, where inhibitory neurons are among the first to be activated. Evidence for such a feedforward response in GABAergic neurons has been shown in culture models [1,3]. A known candidate for this role is the low-threshold spiking (LTS) interneuron, which constitutes 4.5–15% of GABAergic neurons and operates via T-type calcium channels [9,10]. While LTS interneurons have been implicated in feedforward inhibition via T-type calcium channels, this mechanism cannot account for the widespread anticipatory responses we and others have observed in culture [1,3].

In contrast, we propose an alternative mechanism, namely, GABAergic neurons expressing CP-AMPARs and CP-KARs, which, according to our data, represent over half of the GABAergic population in cultures [1]. In our proposed model, CP-KARs on GABAergic neurons are activated at resting potentials. This allows for direct calcium influx through receptors like CP-KARs upon agonist binding, triggering immediate GABA release without requiring prior depolarization. Therefore, we suggest that the predominant mechanism for feedforward inhibition involves CP-KARs, enabling a leading response from the majority of inhibitory neurons and representing a fundamental and widespread mechanism for rapid inhibitory control.

The duration of the delay in glutamatergic neuron excitation varies significantly throughout the population (Figure 2A′). This heterogeneity may be explained by differences in the chloride ion gradient among target neurons, a factor determining whether GABA(A) receptor activation results in hyperpolarization or depolarization [17,18]. Variations in this gradient would lead to differing degrees of GABA(A)R-dependent inhibition, thus accounting for the observed spectrum of delays and potentially contributing to neuronal desynchronization. Furthermore, the specific neural circuit involving the two GABAergic subtypes is critical. We previously showed that GABAergic neurons expressing CP-AMPARs innervate and inhibit those expressing CP-KARs [1,5]. Therefore, when NBQX blocks CP-AMPARs, it likely disinhibits the CP-KAR-expressing GABAergic neurons. This disinhibition increases the release of GABA from these neurons onto their glutamatergic targets, inhibiting them and thereby increasing the excitation delay. (Figure 3A). This mechanism indicates that while both GABAergic subtypes respond early to DoA, the delay in glutamatergic excitation is predominantly controlled by the activity of CP-KAR-expressing neurons. This conclusion is supported by the finding that NBQX, by inhibiting CP-AMPARs to disinhibit CP-KAR-expressing neurons, prolongs the excitation delay (Figure 3A). The delay in glutamatergic excitation is primarily determined by the duration of GABAergic inhibition, as evidenced by its negation with bicuculline. This latency is influenced by several factors, including the timing of GABA reuptake, individual neuronal chloride gradients, and the strength of the concurrent depolarizing current.

Finally, the early-response property of neurons expressing CP-KARs/CP-AMPARs is not specific to DoA. As shown in Figure 4A,C,D, these neurons also respond ahead of the main population to glutamate itself.

To confirm the low-threshold excitability of GABAergic neurons expressing CP-KARs and CP-AMPARs, we applied moderate depolarization (10–12 mV) using NH_4_Cl [28]. As shown in Figure 5A, both neuronal subtypes responded ahead of the general population, a result consistent with previous findings [1,3]. A key characteristic of these receptors is their low conductance, which permits sub-threshold depolarization to elevate intracellular Ca^2+^ levels, triggering GABA release without requiring bursts of AP.

As shown in Figure 6A, NH4Cl induces a biphasic response: an initial hyperpolarization of glutamatergic neurons via GABA release, followed by a slow depolarization likely mediated by GABA removal and the direct depolarizing current of NH_4_Cl. Excitation occurs when this slow depolarization reaches the threshold for sodium and calcium channel activation. Consequently, the net response latency is determined by the balance between the inhibitory GABAergic force and the magnitude of the slow depolarizing current. As this current is agonist-concentration-dependent (Figure 3C), higher concentrations produce a stronger depolarization that overcomes inhibition more rapidly. This explains the inverse relationship between agonist concentration and response delay observed in Figure 3.

We then tested neuronal responses under conditions of enhanced network excitability. Forskolin, a strong activator of adenylate cyclase, increases intracellular cAMP levels. This leads to PKA-dependent phosphorylation, which promotes depolarization by enhancing the activity of Ca^2+^ and Na^+^ channels while suppressing hyperpolarizing K^+^ channels [29]. As expected, forskolin induced strong network-wide activation (increased firing frequency) in mature hippocampal cultures (Figure 5B). Under these conditions, a subset of neurons responded more rapidly. The subsequent application of ATPA (200 nM) induced a [Ca^2+^]_i_ increase specifically in these early-responding cells, identifying them as GABAergic neurons expressing CP-KARs. This rapid response to forskolin resembles the effect of other weak depolarizing agents [22,30], confirming that CP-KAR-expressing neurons are capable of early activation under various excitatory conditions.

To determine if this mechanism of excitation control operates in a more native context, we performed experiments on hippocampal slices obtained from two-month-old animals. Figure 7 demonstrates that a small population of neurons in these slices exhibited a leading response to both depolarization and DoA, a result analogous to our culture findings. The same neurons responded to both stimuli earlier than other neurons, indicating the presence of an anticipatory GABAergic mechanism in vivo.

Consequently, our data reveal that a subset of GABAergic neurons expressing CP-KA and CP-AMPA receptors generate a pre-emptive inhibitory signal in response to hyperexcitability, preventing glutamatergic network synchronization. This anticipatory response represents a previously unrecognized mechanism for maintaining circuit stability.

## Data Availability

The original contributions presented in this study are included in the article. Further inquiries can be directed to the corresponding author.

## Supplementary Materials

The following supporting information can be downloaded at: https://www.mdpi.com/article/10.3390/cimb48010082/s1. Reference [5] is cited in the Supplementary Materials.

## Abbreviations

GABA	γ-aminobutyric acid
DIV	days in vitro
AP	action potential
DoA	domoic acid
ATPA	calcium-permeable kainate receptor agonist, (RS)-2-amino-3-(3-hydroxy-5-tertbutylisoxazol-4-yl) propanoic acid
CP-KARs	calcium-permeable kainate receptors
CP-AMPARs	calcium-permeable AMPA receptors
