# Supplementary material for: The Role of Calcium-Permeable Kainate and AMPA Receptors in the Leading Reaction of GABAergic Neurons to Excitation
Source: Curr Issues Mol Biol. 2026 Jan 14;48(1):82. doi: 10.3390/cimb48010082 (PMC12839610; doi:10.3390/cimb48010082)
Supplement: Supplementary file 1 [file cimb-48-00082-s001.zip › Figure S1.pdf]

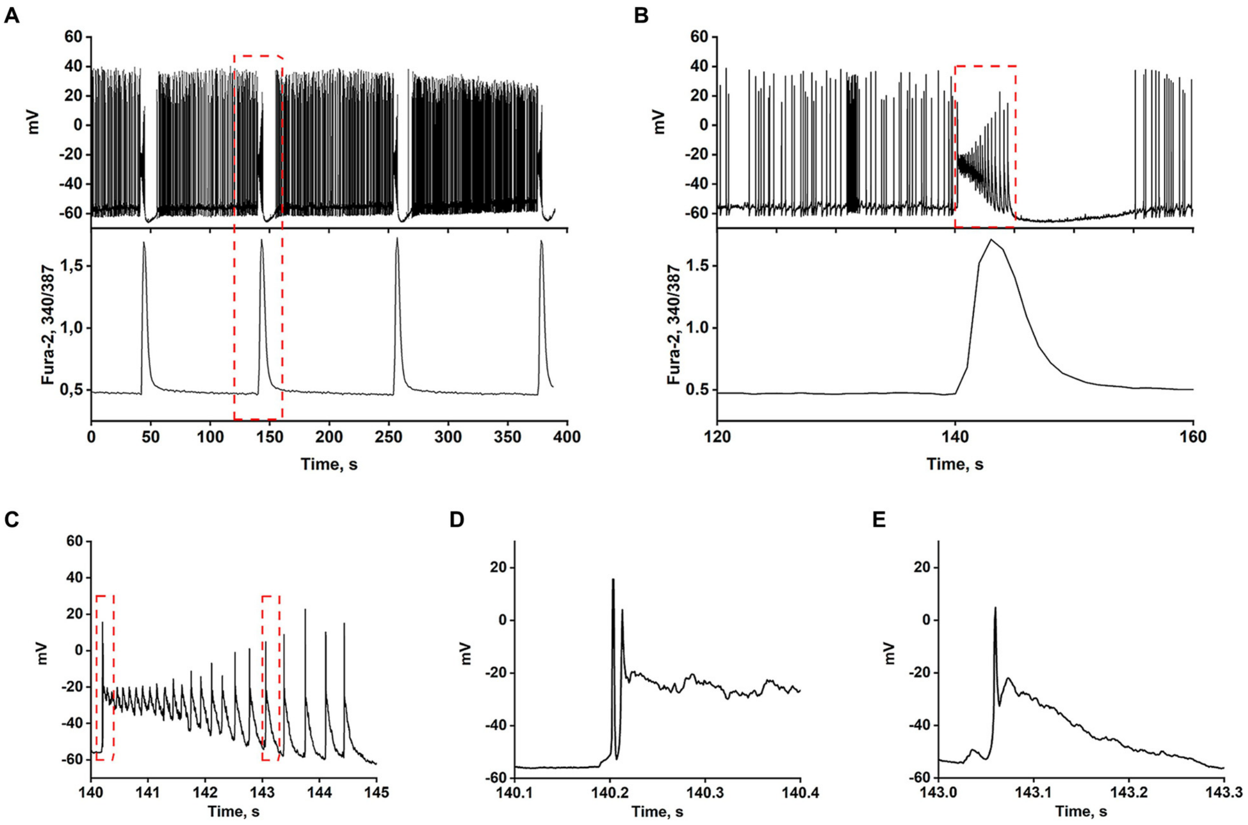

Figure S1. Correlation of membrane potential burst activity and Ca2+ pulses. In the presence of bicuculline (10 μM), the spontaneous tonic activity of AP is periodically interrupted by burst activity accompanied by a Ca^{2+}-pulse. (**A**) Changes in membrane voltage (top) and $[Ca^{2+}]_i$ (bottom) of a neuron. (**B**) Representative PDS cluster; changes in membrane potential (top panel) and $[Ca^{2+}]_i$ (bottom panel). (**C**) The magnified PDS cluster from Figure part label (**B**). (**D**) The magnified leading edge of the cluster from Figure part label (**C**). (**E**) One of the individual PDSs from Figure part label (**C**). The AP is generated at the leading edge. The duration of the PDS is ~300 ms [1].

Reference

1. Zinchenko, V.P.; Teplov, I.Y.; Kosenkov, A.M.; Gaidin, S.G.; Kairat, B.K.; Tuleukhanov, S.T. Participation of calcium-permeable AMPA receptors in the regulation of epileptiform activity of hippocampal neurons. *Front. Synaptic Neurosci.* **2024**, *16*, 1349984.
